# ﻿A new species of *Esola* Edwards, 1891 (Crustacea, Copepoda, Harpacticoida, Laophontidae) from the Caribbean coast of Colombia

**DOI:** 10.3897/zookeys.1074.73030

**Published:** 2021-12-01

**Authors:** Juan Manuel Fuentes-Reinés, Eduardo Suárez-Morales, Marcelo Silva-Briano

**Affiliations:** 1 El Colegio de la Frontera Sur, Apartado postal 424, 77014 Chetumal, Quintana Roo, Mexico Universidad del Magdalena Santa Martha Colombia; 2 Universidad del Magdalena, Grupo de Investigación en Biodiversidad y Ecología Santa Marta, Magdalena, A.A. 731,Colombia El Colegio de la Frontera Sur Chetumal Mexico; 3 Universidad Autónoma de Aguascalientes, Centro de Ciencias Básicas, Ciudad Universitaria, Aguascalientes 2013, Mexico Universidad Autónoma de Aguascalientes Aguascalientes Mexico

**Keywords:** Benthic copepods, Caribbean, crustaceans, harpacticoids, taxonomy

## Abstract

A new species of the harpacticoid copepod genus *Esola* is described from specimens collected in Rodadero Beach, on Gaira Bay, on the Caribbean coast of Colombia. The species, *E.wellsi***sp. nov.**, is described, illustrated, and com­pared with its congeners. *Esolawellsi***sp. nov.** differs from its known congeners in details of the armature of legs 1–4. It most closely resembles *E.bulbifera* (Norman, 1911) in the armature formula of P1–P5 but differs from the latter in several respects, including the female antennule segmentation (7-segmented in *E.bulbifera* but distinctly 6-segmented in *E.wellsi***sp. nov.**) and in the shape and size of the male P3ENP2 apophysis, among other characters. This is the second species of the genus known from the Caribbean and the second record of *Esola* in the Northwestern Tropical Atlantic. The genus now contains eight species. A key to the known species of the genus is also included.

## ﻿Introduction

The family Laophontidae is one of the largest in the copepod order Harpacticoida; it contains over 320 species and 63 genera. Huys and Lee (2000) subdivided the family into two subfamilies: Esolinae and Laophontinae, with the latter containing 95% of the known laophontid species (Boxshall and Halsey 2004).

Most laophontids are commonly found as benthic forms living in costal marine and transitional environments (Huys and Lee 2000; Boxshall and Halsey 2004), but some genera can be found in fully freshwater habitats (Defaye and Dussart 2011). Laophontids have been known to occur in various types of sediments and benthic habitats including interstitial (i.e., *Afrolaophonte* Chappuis, 1960) (Boxshall and Halsey 2004), mangroves (i.e., *Echinolaophonte* Nicholls, 1941) (Willey 1930), coral substrates (i.e., *Peltidiphonte* Gheerardyn and Fiers 2006) (Gheerardyn et al. 2006), as symbionts of marine invertebrates (Cottarelli et al. 2006; Cottarelli and Bruno 2011), and some genera (e.g., *Bathylaophonte* Lee & Huys, 1999 and *Bathyesola* Huys & Lee, 2000) have been reported from deep-sea habitats (Boxshall and Halsey 2004).

The genus *Esola* Edwards, 1891 is considered to be cosmopolitan (Huys and Lee 2000) and has been recorded from various localities of Europe (i.e., Ligurian Sea, Scotland, British Isles, Ireland; Norfolk, Mediterranean Sea, Corsica), Africa (Suez Canal, Kenya), Asia, Oceania (Ifaluk Atoll, Caroline Islands), and the Americas (Galapagos, Brazil, Bahamas). According to Suárez-Morales et al. (2006) laophontids are represented by 21 species and 11 genera in the Caribbean Sea. Currently, *Esola* is known to contain seven nominal species (Wells 2007): *E.longicauda* Edwards, 1891; *E.bulbifera* (Norman, 1911); *E.galapagoensis* Mielke, 1981; *E.canalis* Huys & Lee, 2000; *E.lobata* Huys & Lee, 2000; *E.profunda* Huys & Lee, 2000, and *E.vervoorti* Huys & Lee, 2000. Only one species, *E.longicauda* from Bahamas has been recorded in the Americas and the Northwestern Tropical Atlantic (NWTA) region (Reid 1998; Huys and Lee 2000).

The knowledge on this harpacticoid family is practically non-existent for Colombian waters; a recent biological survey of the crustacean fauna of Rodadero Beach on Gaira Bay, a large embayment on the Caribbean coast of Colombia, yielded several male and female specimens of laophontid harpacticoid copepods. Some of these specimens were taxonomically examined and found to represent an undescribed species of *Esola*. The new species is here described, illustrated, and com­pared with its known congeners.

## ﻿Materials and methods

Biological samples of littoral habitats were obtained from Rodadero Beach, Gaira Bay, Magdalena, northern Colombia (11°12'30.120"N, 74°13'39.13"W) during fieldwork carried out from August 2015 to March 2016, mainly at inshore areas covered by mangrove vegetation and in an adjacent oyster bank. Water salinity, pH, and temperature were measured at each sampling site with the aid of a WTW 350i multiparameter equipment. Water samples were collected manually using a 25-L bucket at both littoral and limnetic habitats. Samples were filtered with a zooplankton net (mesh size = 45 μm) and preserved in 70% ethanol. Copepods were sorted from all the samples and then processed for taxonomic identification, including the examination of the whole specimen and the dissection of selected appendages. Dissected appendages were mounted on semi-permanent slides with glycerine and sealed with Canada balsam. Specimens were measured in ventral position, from the anterior end of the rostral area to the posterior margin of the caudal rami. Drawings were made with the aid of a camera lucida mounted on an Olympus BX51 compound microscope equipped with Nomarski DIC. Two female individuals were prepared for SEM examination with a JEOL LV 5900 microscope at the University of Aguascalientes (**UAA**), Mexico. The process included dehydration of specimens in progressively higher ethanol solution (70–100%), critical point drying, and gold coating following standard methods. The type specimens were deposited at the Centro de Colecciones Biológicas de la Universidad del Magdalena, Colombia (**CBUMAG**), where they are available for consultation and/or further examination.

The morphologi­cal terminology of the description followed Huys and Boxshall (1991). The abbreviations used in the descriptive section are: **P1–P6** = first to sixth legs, **EXP** = exopod, **ENP** = endopod, **s** = setae, **ae** = aesthetascs.

## ﻿Results

### ﻿Taxonomy

#### Order Harpacticoida G.O. Sars, 1903

**Family Laophontidae T. Scott, 1904**

##### Genus *Esola* Edwards, 1891

###### ﻿ Esola wellsi sp. nov.

Taxon classificationAnimaliaHarpacticoidaLaophontidae

76711CAE-00E1-5BA8-9DA4-CBA67C3FF66F

http://zoobank.org/B924D013-ABE9-4ADF-8E90-CBB3565E2DFA

**Figure 1.**
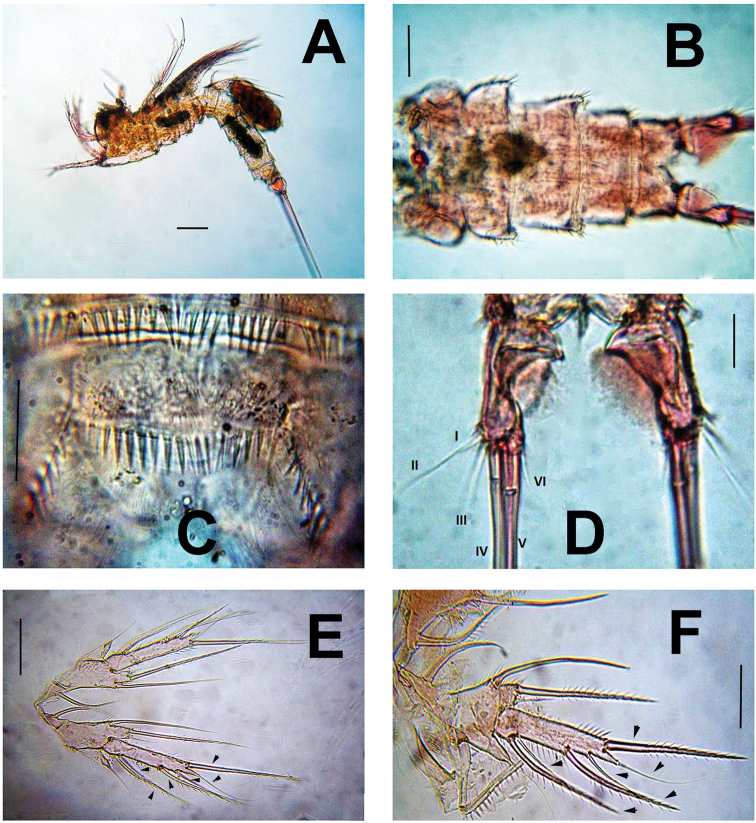
*Esolawellsi* sp. nov., adult female from Colombia **A** habitus, lateral view **B** urosome, ventral view **C** anal operculum **D** caudal rami, ventral view **E** fifth leg **F** same, another specimen. Scale bars: 100 μm (**A**); 50 μm (**B, C**); 20 μm (**D**).

**Figure 2.**
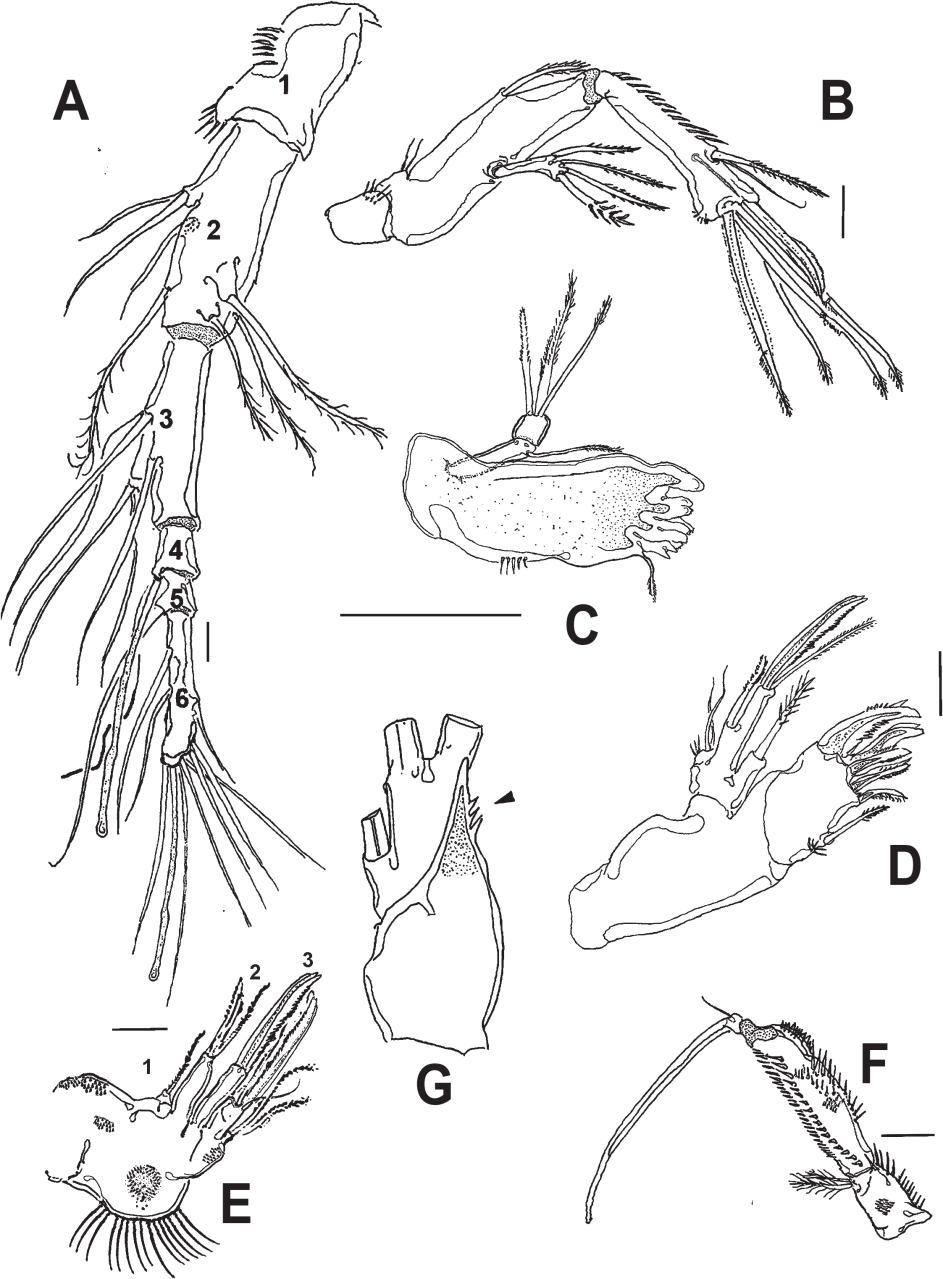
*Esolawellsi* sp. nov., adult female from Colombia **A** antennule **B** antenna **C** mandible with palp **D** maxillule **E** maxilla **F** maxilliped **G** adult male from Colombia, P3 ENP pectinate apophysis (arrow). Scale bars: 10 μm (**A–G**).

**Figure 3.**
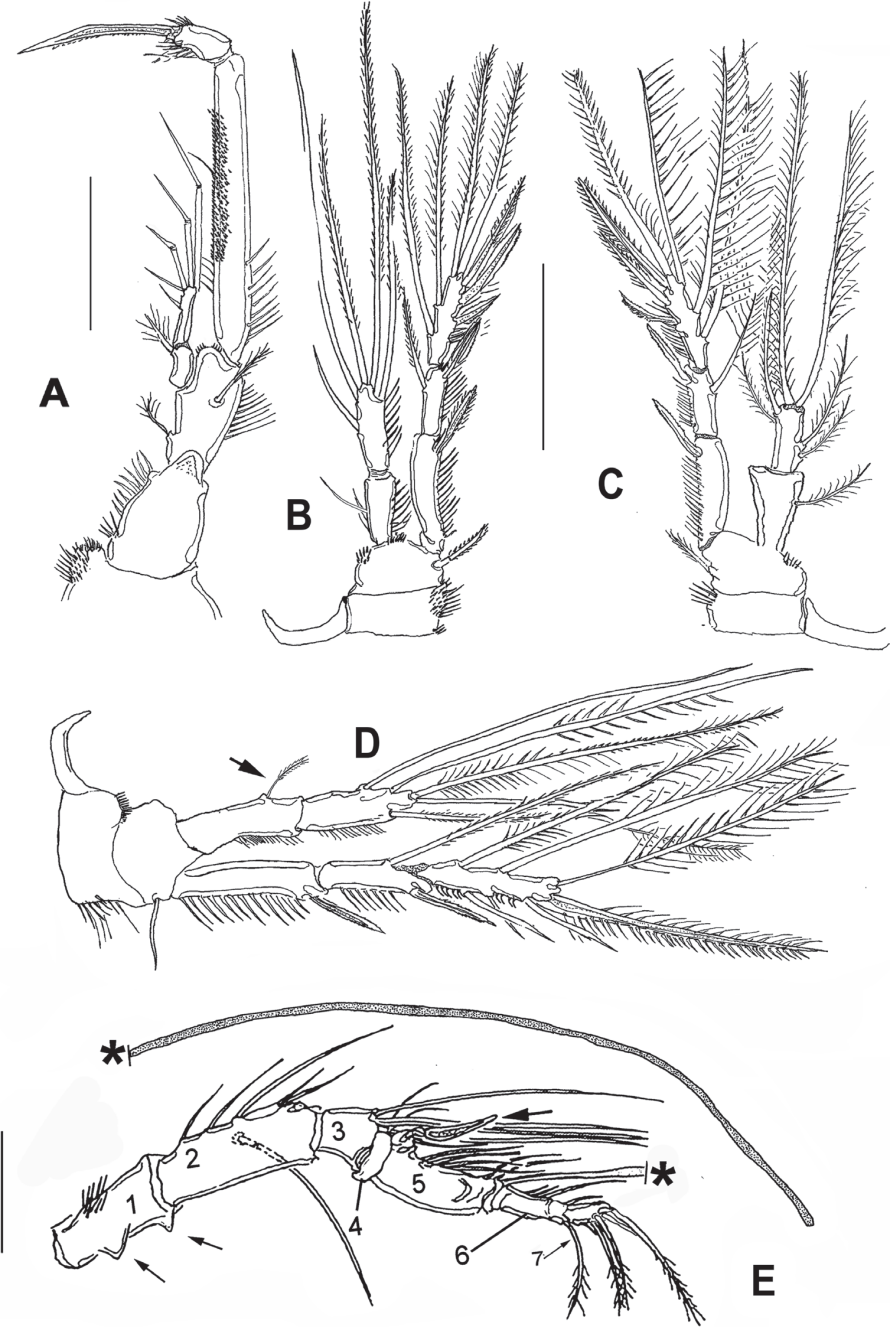
*Esolawellsi* sp. nov., adult female from Colombia **A** leg 1 **B** leg 2 **C** leg 3 **D** leg 4 **E** adult male from Colombia, geniculate antennule with long aesthetasc (*). Scale bars: 50 μm (**A**); 100 μm (**B–D**); 25 μm (**E**).

**Figure 4.**
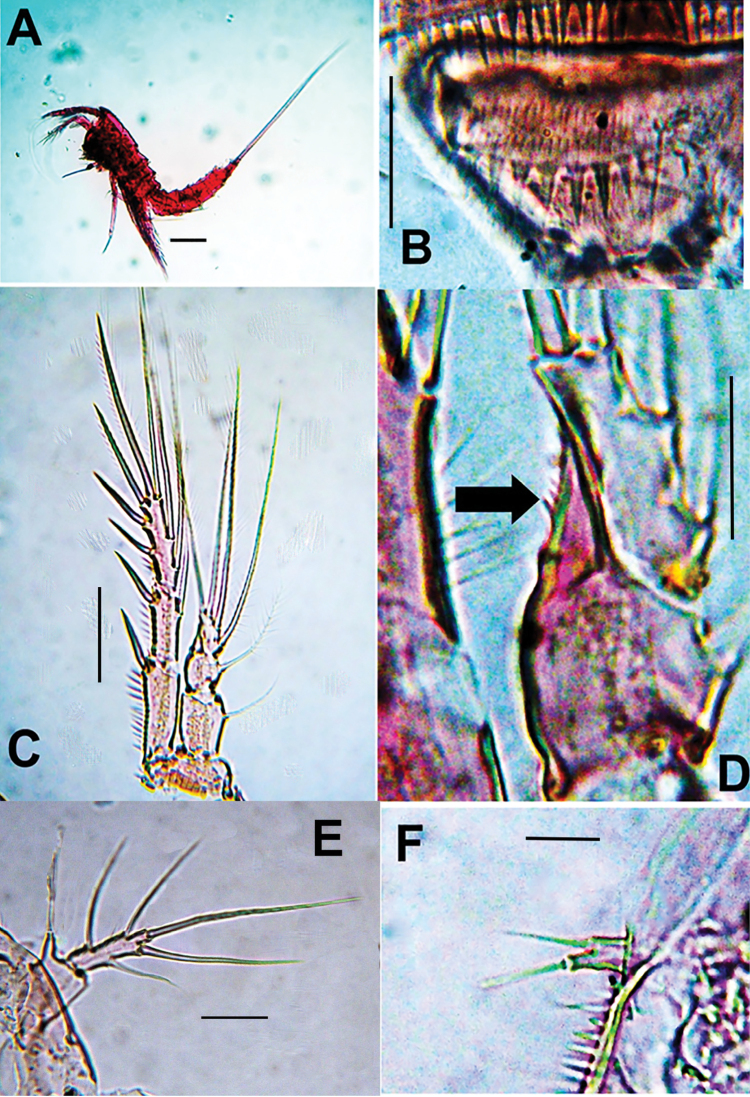
*Esolawellsi* sp. nov., adult male from Colombia **A** habitus, lateral view **B** anal operculum, dorsal view **C** P3 **D** P3 ENP dentate apophysis (arrowed) **E** leg 5 **F** sixth leg. Scale bars: 100 μm (**A**); 50 μm (**B, C, E**); 20 μm (**D, E**); 10 μm (**F**).

**Figure 5.**
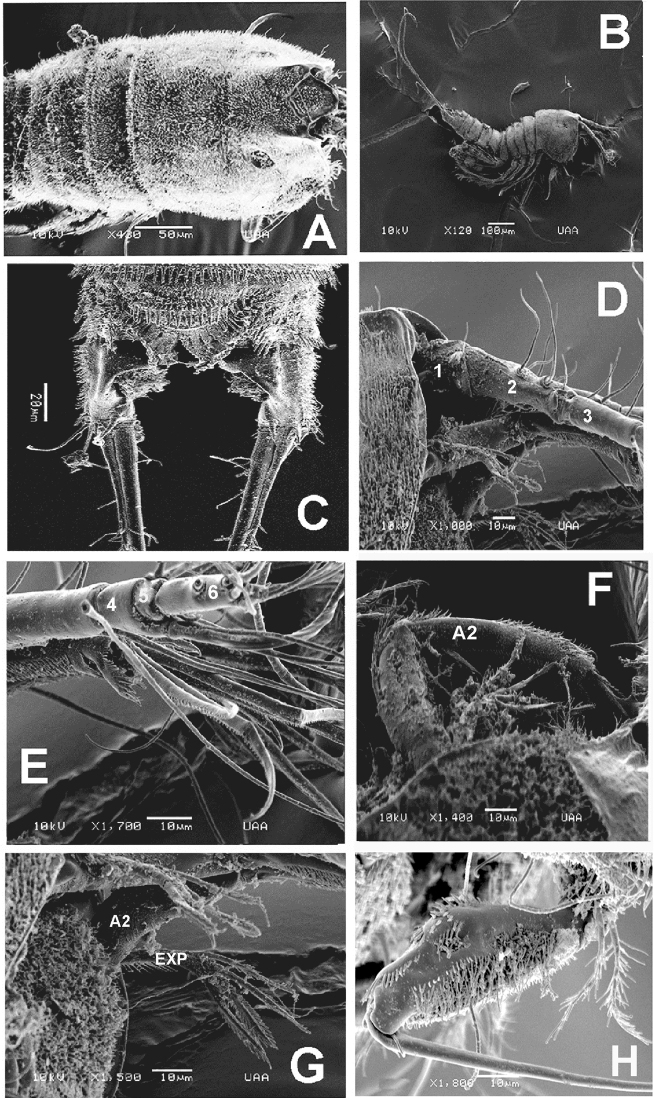
*Esolawellsi* sp. nov., adult female from Colombia, SEM-prepared specimen **A** densely spinulate prosome, dorsal view **B** habitus, lateral view **C** anal somite dorsal view **D** antennule segments 1–3 **E** same, segments 4–6 **F** antenna (A2) **G** antenna (A2) showing exopod (EXP) **H** maxilliped.

**Figure 6.**
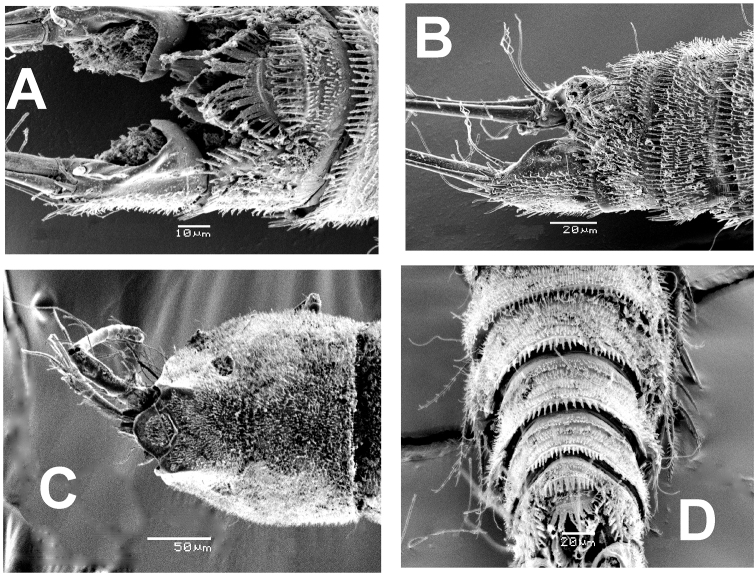
*Esolawellsi* sp. nov., adult female from Colombia, SEM-prepared specimen **A** anal somite, caudal rami and ornamentation of operculum dorsal view **B** same, semi-lateral view **C** cephalosome, dorsal view **D** urosome dorsal view showing ornamentation of urosomites distal margins.

**Figure 7.**
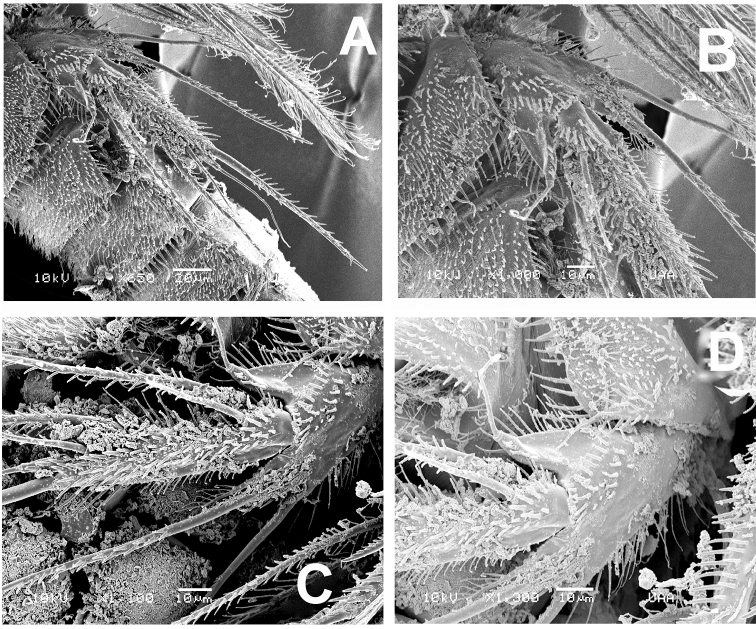
*Esolawellsi* sp. nov., adult female from Colombia, SEM-prepared specimen **A** proximal urosomites semi-lateral view and leg 5 **B** leg 5, ventral view **C** same, lateral view **D** fifth leg, detail of leg 5 EXP insertion.

Figures 1 , 2 , 3 , 4 , 5 , 6 , 7

####### Type material.

Adult female holotype (CBUMAG: MEI: 0034), male allotype (CBUMAG: MEI: 0036) from Rodadero Bay, Magdalena, northern Colombia, coll. J. Fuentes-Reinés, August 2015–June 2016. Paratypes: 7 females (CBUMAG: MEI: 0035) and 4 males (CBUMAG: MEI: 0037) same locality and collector. Two adult females processed for SEM examination.

####### Type locality.

Rodadero Beach, Gaira Bay, Magdalena, northern Colombia (11°14'10"N, 74°12'06"W).

Water temperature at this mangrove site varies seasonally between 30 and 32 °C, salinity is 36.1 psu, and pH is 8.3.

####### Etymology.

The species is named after Dr John B. Wells as a tribute for his longstanding, solid contributions to the taxonomic knowledge of harpacticoid copepods (Grieve et al. 2019; Huys 2021). It is a noun in apposition, gender masculine.

####### Differential diagnosis.

With characters of laophontid genus *Esola*, body covered by dense pattern of small spinules, closely resembling *E.bulbifera* as redescribed by Huys and Lee (2000) in most respects, including body length and armature of legs 1–4, but female antennule distinctly 6-segmented, male geniculate antennule 7-segmented, subchirocer. Male P3 apophysis distinctively pectinate.

####### Description of female.

Habitus (Figs 1A, 5B). Body roughly cylindrical in dorsal view, pro­some gradually tapering anteriorly. Total body length measured from anterior margin of rostrum to posterior margin of caudal rami ranging from 616 to 658 μm (average length = 640 μm, *n* = 8; holotype length = 658 μm).

Rostrum triangular in lateral view, rounded in dorsal view (Figs 5A, 6C). Genital double-somite with dorsal suture, somite completely fused ventrally (Fig. 1B). Cephalothorax slightly wider than free prosomites, with anterodorsal row of spinules. Cephalosome with dense pattern of small spinules (Figs 5A, 6C). Ventral surface of urosome smooth except for penultimate urosomite furnished with row of spinules along posterior margin. Urosomites (Figs 6B, D, 7A) with dense pattern of dorsal and lateral small spinules. Anal somite short, with rounded anal operculum furnished with 12–16 large spinules (Figs 5C, 6A).

Caudal rami (Figs 1D, 5C, 6A, B) subrectangular, length/width ratio = 2.6. Proximal half distinctly expanded, distal half narrow, concave. Outer margin furnished with spinules. Rami with 7 setal elements. Seta I small, spiniform. Seta II and III smooth and closely set, almost equally long, seta V about twice as long as seta IV, both lightly pinnate and with spinules at insertion point. Seta VI about ½ length of seta VII.

Antennule (Figs 2A, 5D, E) 6-segmented. First segment with rows of small spinules along outer and distal margins, second segment longest, cylindrical, about 3.3 times as long as wide; third segment slender, about 3.4 times as long as wide, armed with aesthetasc fused with 1 smooth seta). Acrothek set on apical pedestal. Segmental antennule armature as follows, s = setae, ae = aesthetascs: 1(1s)-2(8s)-3(6s)-4(2+ae)-5(1s)-6(9+acrothek).

Antenna (Figs 2B, 5F, G) 3-segmented, comprising coxa, allobasis, free 1-segmented ENP and 1-segmented EXP. Coxa small, with row of spinules. Allobasis elongate, with pinnate abexopodal seta inserted near distal margin. EXP slender, about 5 times longer than wide, with 4 well-developed pinnate setae (2 lateral, 2 apical). ENP slightly longer than allobasis, subquadrate, 1.1 times as long as wide, with row of spinules on inner and outer margins; lateral armature arising from distal half, consisting of small, slender seta flanked by 2 pinnate spines. Subdistal part with thick spine; apical armature consisting of 4 setae.

Mandible (Fig. 2C) with gnathal blade narrow, bearing several multicuspidate teeth plus short pinnate seta. Palp slender 2-segmented, first segment (basis) cylindrical, armed with single seta; second segment (endopod) subquadrate, with 3 subequally long apical setae.

Maxillule (Fig. 2D). Precoxal arthrite with 8 distal spines/setae, inner margin with single pinnate seta. Coxa with cylindrical endite carrying slender seta. Basal endite cylindrical, unfused, armed distally with 2 setae and 1 spine. Endopod incorporated in basis, forming small peduncle armed with single seta. Exopod 1-segmented, with plumose apical seta.

Maxilla (Fig. 2E). Syncoxa with row of long slender spinules on outer margin plus rounded smooth protuberance. Three armed syncoxal endites (1–3 in Fig. 2F) including small first endite, with single seta, second and third endites each armed with 2 stout elements. Allobasis transformed into strong, slightly curved, distally pinnate claw with single proximal slender seta. Endopod represented by 2 setae.

Maxilliped (Figs 2F, 5H) slender, represented by cylindrical syncoxa with proximal patch of small spinules and short setules along outer margin, segment armed with 2 equally long distal plumose setae. Basis with rows of small, strong spinules along inner margin plus small spinule patches on medial and subdistal position of outer margin, basis about 1.4 times as long as syncoxa. ENP short, forming long, curved claw. Slender seta inserted at base.

P1 (Fig. 3A) with dense cuticular ornamentation on precoxa, coxa, and basis. Basis with short pinnate seta on anterior surface; with outer basipodal seta and distal seta, surface furnished with spinules. EXP short, 2-segmented, reaching proximal ¼ of ENP1 length. EXP1 with 1 outer spine, EXP2 with 2 outer spines and 1 outer geniculate seta, and 2 apical geniculate setae. ENP1 about 8 times as long as wide, ENP2 short, 1.6 times as long as wide, with long distal claw and slender accessory seta. Intercoxal sclerite narrow, smooth.

P2 (Fig. 3B) Coxa furnished with long spinules along outer margin. Basis with short bipinnate outer spine. ENP 2-segmented, shorter than EXP, almost reaching insertion of EXP2 inner seta; ENP1 with 1 inner seta, ENP2 with 2 inner, 2 apical, and 1 outer seta. EXP 3-segmented, segments with outer spinules; EXP1 smooth, EXP2 with inner seta, EXP 3with 7 elements (3 outer spines, 2 apical setae, 2 inner elements). Intercoxal sclerite narrow, smooth.

P3 (Fig. 3C) Coxa as in P2. Basis with lightly plumose outer seta. ENP 2-segmented, shorter than EXP, not reaching insertion of inner seta of P3EXP2; ENP 1 with single inner seta, ENP 2 with 6 elements, 2 inner, 2 apical, and 2 outer setae. EXP 3-segmented, outer margin of segments furnished with spinules; EXP 1 armed with outer spine, inner margin smooth; EXP 2 with inner seta; EXP3 armed with 7 elements (3 outer spines, 2 apical setae, 2 inner setae). Intercoxal sclerite narrow, smooth.

P4 (Fig. 3D) Coxa and basis as in P3. ENP 2-segmented, shorter than EXP, reaching tip of EXP2; ENP 1 with short, slender inner seta arrowed in Fig. 3D), ENP2 with 5 elements (2 inner, 2 apical, 1 outer). EXP 3-segmented, segments furnished with small spinules on outer margins (Fig. 6F). EXP1 lacking inner seta, EXP 2 with inner seta; EXP3 with 7 elements (3 outer spines, 2 apical setae, 2 inner elements). Intercoxal sclerite narrow, smooth.

Armature formula P2–P4

P5 (Figs 1E, F, 7A–D) slender, with EXP and baseoendopod covered with small spinules. Setophore armed with long seta. Endopodal lobe short, reaching insertion of outer seta of EXP, with 1 short and 1 long pinnate setae apically, plus 2 long inner setae. EXP elongate, about 6 times as long as wide, armed with 6 elements (each arrowed in Fig. 1E, F).

####### Description of male.

Habitus (Fig. 4A) as in female except for urosome segmentation. Total body length ranging from 476 to 518 μm (average = 492 μm; *n* = 10; allotype = 476 μm).

Antennule (Fig. 3E) 7-segmented, subchirocer. Geniculation between segments 5 and 6. Segment 1 with row of spinules/setules on anterior and distal margins, with 2 spinous processes on inner margin (arrows in Fig. 3E). Segment 2 longest; segment 4 shortest; segment 5 bearing strong spiniform process proximally (arrowed in Fig. 3E) and very long distal aesthetasc. Segment 7 with 6 apical setae plus acrothek. Armature formula as follows: 1(1s)-2(9s)-3(4s)-4(2s)-5(7s, 1 pinnate + 1 spine + 2s + ae)-6(1s + 2process)-7(6s+ acrothek).

Mouthparts, P1, P2 and P4 as in female.

P3 (Figs 2G, 4C, D) with 3-segmented EXP and ENP. EXP as in female. ENP1 and ENP2 with inner seta, ENP2 bearing distinctively dentate apophysis (arrowed in Figs 2G, 4D), ENP3 with 2 lateral and 2 apical setae.

P5 (Fig. 4E) with basoendopod lacking endopodal lobe. EXP relatively smaller than in female, about 3.6 times as long as wide, armed with 5 setae (2 outer pinnate, 1 plumose apical, 1 pinnate inner distal, and 1 smooth inner proximal).

P6 (Fig. 4F) represented by small subrectangular segment armed with 1 small lateral and subequally long apical seta.

Anal operculum (Fig. 4B) stronger than in female, with 8–10 large spinules and small spinules on surface. Caudal ramus subrectangular, lacking bulbiform expansions as those observed in the female. Distal part about 1.5 times as long as wide.

####### Remarks.

Our specimens from Colombia were identified as members of the genus *Esola* by their possession of the generic characters proposed by Huys and Lee (2000), including: 1) body cylindrical, 2) genital double-somite and third urosomite laterally produced, 3) anal operculum spinulose, 4) caudal rami expanded, forming bulbous process dorsally, 5) sexual dimorphism in antennules, P3ENP, P5, and caudal rami, 6) female antennule 6-segmented, subchirocer, male antennule 7-segmented, 7) antennary exopod with 4 stout setae, 8) 2-segmented mandibular palp, 9) maxilliped slender, syncoxa armed with 2 setae, 10) P1EXP and ENP 2-segmented, EXP2 with 4 or 5 setae, ENP1 longer than ENP2, 11) P2–P4 EXP and ENP with 3 and 2 segments, respectively, 12) P5 ENP and EXP with 4 and 6 setae, respectively, and EXP longer than ENP.

The new species of *Esola* can be distinguished from its known congeners by a combination of characters including: 1) a relatively robust body; 2) female body length ranging from 616 to 630 µm; and 3) robust caudal rami. The most important characters of *E.wellsi* are: 1) mandibular palp with 4 setae 2) P1EXP2 with 3 geniculate setae, 3) P2 and P4 ENP1 with inner seta, 4) caudal rami length/distal width ratio = 2.0, and 5) outer apical length seta on female P5BENP short.

We followed Wells (2007) in identifying a group of morphologically similar species of *Esola* among which we compared *E.wellsi* sp. nov. According to our analysis, and following Wells (2007), the setal formula of the P2–4EXP3 (6:7:7), 2) the absence of an inner seta on P2–P4EXP1 (1–1–1), and the number of setae on P2–P4ENP (5–6–5) of *E.wellsi* sp. nov. are shared with several congeners: *E.profunda*, *E.lobata*, *E.bulbifera*, *E.canalis*, *E.longicauda*, and *E.galapagoensis*.

Overall, the new species most closely resembles *E.bulbifera* in the armature formula of P1–P5 and the armature of the mandibular palp. These two species can be distinguished by the following characters: 1) female antennule indistinctly 7-segmented in *E.bulbifera* versus distinctly 6-segmented in *E.wellsi* sp. nov. (compare Figs 2A, 5D, E with fig. 2C in Huys and Lee 2000), 2) P1ENP2 is more robust and relatively shorter in *E.wellsi* sp. nov. than in *E.bulbifera* (compare Fig. 3A with fig. 3A in Huys and Lee 2000), 3) the P1ENP2 terminal claw is 3.5 times as long as ENP2 in *E.wellsi* sp. nov. versus 2.4 times in *E.bulbifera* (compare Fig. 3A with fig. 3E in Huys and Lee 2000), 4) the first exopodal segment of P1EXP1 reaches the insertion of P1ENP in *E.wellsi* sp. nov. whereas in *E.bulbifera* it does not reach this level (compare Fig. 3A with fig. 3A in Huys and Lee 2000), 5) the female P5 ENP outer seta is relatively shorter in *E.wellsi* sp. nov. than in *E.bulbifera* (compare Fig. 1F with fig. 3D in Huys and Lee 2000), 6) the spiniform process on the male antennulary segment 5 is simple and spiniform in *E.wellsi* sp. nov. versus bifid in *E.bulbifera* (compare Fig. 3E with fig. 5D in Huys and Lee 2000), 7) the male P3ENP2 of *E.wellsi* sp. nov. bears a dentate apophysis versus a simple process in *E.bulbifera* (compare Figs 2G, 4D with fig. 4D in Huys and Lee 2000), 8) the male P3ENP2 apophysis does not reach the distal margin of P3ENP3 in *E.wellsi* sp. nov. versus a process extending beyond distal margin of P3ENP3 as shown by *E.bulbifera* (compare Figs 2G, 4D with fig. 4D in Huys and Lee 2000), 9) the male P5 bears 2 outer elements in *E.wellsi* sp. nov. versus 3 outer setal elements in *E.bulbifera* (compare Fig. 4E with fig. 5G in Huys and Lee 2000, 10) length/width ratio of male P5 EXP = 4.6 in the new species versus 4.3 in *E.bulbifera*, 11) the female caudal rami length/width ratio = 2.6 in *E.wellsi* sp. nov. (Figs 5C, 6A) versus 2.3 in *E.bulbifera* (table 1 in Huys and Lee 2000), and 12) the dorsal caudal seta VII is as long as ramus in *E.wellsi* sp. nov. versus shorter than ramus in *E.bulbifera* (compare Fig. 6B with fig. 1D in Huys and Lee 2000). These differences are considered sufficient to justify the proposal of a new species of *Esola*.

####### Distribution and ecology.

The new species is currently known only from the type locality, Rodadero Bay, Caribbean coast of Colombia. It was found in a mangrove system at a depth of 0.70 m, where the water temperature varies seasonally between 30 and 32 °C, salinity is 36.1 psu, and pH is 8.3. It is likely that it has a wider distributional range in similar habitats of the western Caribbean region.

### ﻿Key to the known species of *Esola* (females)

**Table:** 

1	P1EXP2 with 4 setae	**2**
–	P1EXP2 with 5 setae	**3**
2	P4ENP1 without seta	***E.galapagoensis* Mielke, 1981**
–	P4ENP1 with seta	***E.longicauda* Edwards, 1891**
3	P2-P3ENP1 without inner seta	***E.vervoorti* Huys & Lee, 2000**
–	P2-P3ENP1 with inner seta	**4**
4	P4ENP1 with vestigial seta	**5**
–	P4ENP1 without seta	***E.lobata* Huys & Lee, 2000**
5	Mandibular palp 2-segmented	**6**
–	Mandibular palp 1-segmented	***E.bulbifera* (Norman, 1911)**
6	P4ENP1 with inner seta	**7**
–	P4ENP1 without inner seta	***E.profunda* Huys & Lee, 2000**
7	Mandibular palp with 5 elements, first segment of mandibular palp with 2 setae, caudal rami moderately expanded, about 3 times longer than wide	***E.canalis* Huys & Lee, 2000**
–	Mandibular palp with 4 elements, first segment of mandibular palp with 1 seta, caudal rami proximal half strongly expanded, about 2.6 times as long as wide	***E.wellsi* sp. nov.**

## Supplementary Material

XML Treatment for Esola wellsi
